# Supplementary figures and images for: Genomic Rewilding of Domestic Animals: The Role of Hybridization and Selection in Wolfdog Breeds
Source: Genes (Basel). 2025 Jan 19;16(1):102. doi: 10.3390/genes16010102 (PMC11764532; doi:10.3390/genes16010102)

# a-score optimisation – spline interpolation

Optimal number of PCs: 7

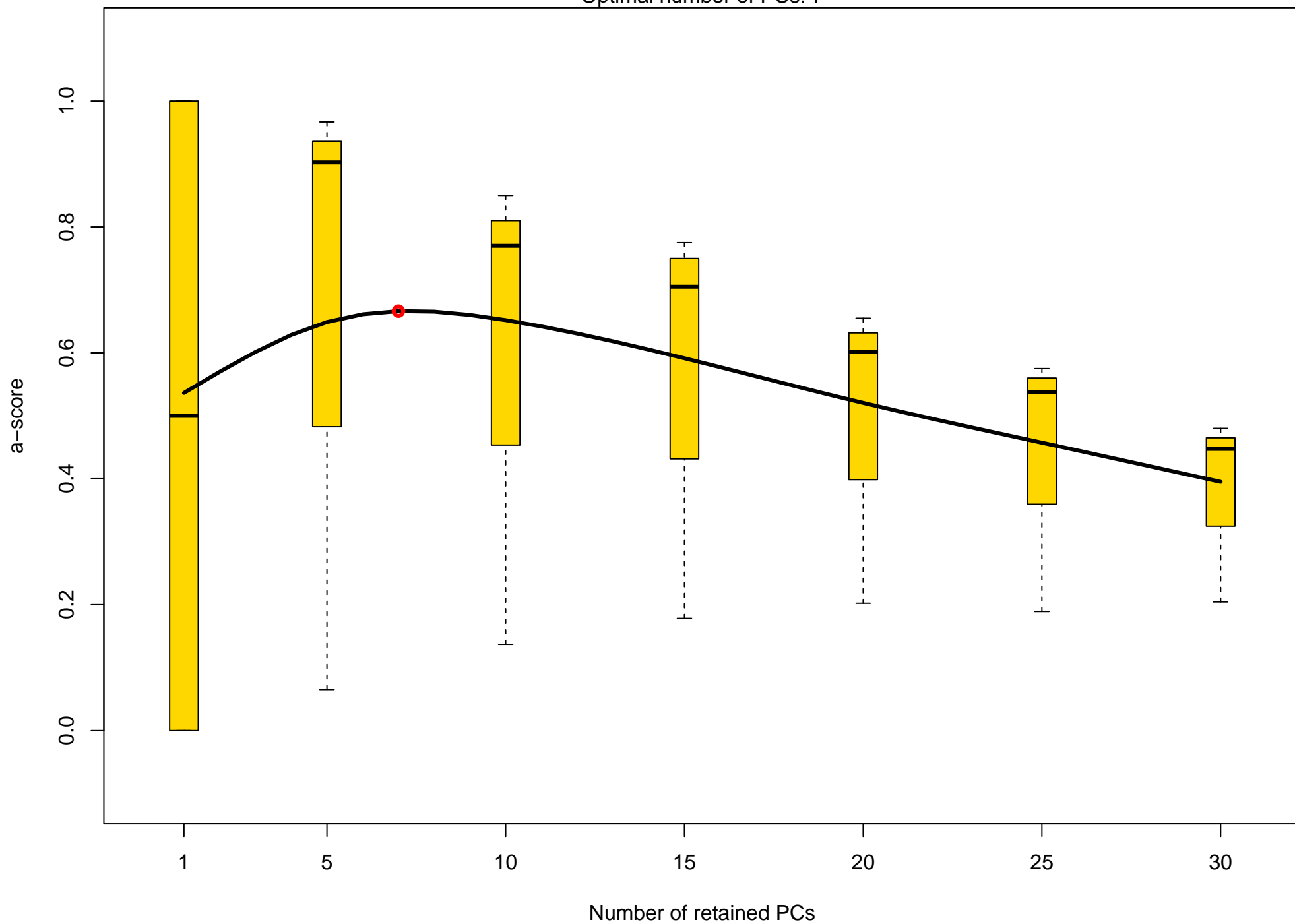

Supplement: Supplementary file 1 [file genes-16-00102-s001.zip › Figure S1 Optimal number of axes.pdf]
